# Establishing Zebrafish as a Novel Exercise Model: Swimming Economy, Swimming-Enhanced Growth and Muscle Growth Marker Gene Expression

**DOI:** 10.1371/journal.pone.0014483

**Published:** 2010-12-31

**Authors:** Arjan P. Palstra, Christian Tudorache, Mireia Rovira, Sebastiaan A. Brittijn, Erik Burgerhout, Guido E. E. J. M. van den Thillart, Herman P. Spaink, Josep V. Planas

**Affiliations:** 1 Departament de Fisiologia, Facultat de Biologia, Institut de Biomedicina de la Universitat de Barcelona (IBUB), Universitat de Barcelona, Barcelona, Spain; 2 Sylvius Laboratory, Molecular Cell Biology, Institute of Biology, Leiden University (IBL), Leiden, The Netherlands; University of Las Palmas de Gran Canaria, Spain

## Abstract

**Background:**

Zebrafish has been largely accepted as a vertebrate multidisciplinary model but its usefulness as a model for exercise physiology has been hampered by the scarce knowledge on its swimming economy, optimal swimming speeds and cost of transport. Therefore, we have performed individual and group-wise swimming experiments to quantify swimming economy and to demonstrate the exercise effects on growth in adult zebrafish.

**Methodology/Principal Findings:**

Individual zebrafish (n = 10) were able to swim at a critical swimming speed (U_crit_) of 0.548±0.007 m s^−1^ or 18.0 standard body lengths (BL) s^−1^. The optimal swimming speed (U_opt_) at which energetic efficiency is highest was 0.396±0.019 m s^−1^ (13.0 BL s^−1^) corresponding to 72.26±0.29% of U_crit_. The cost of transport at optimal swimming speed (COT_opt_) was 25.23±4.03 µmol g^−1^ m^−1^. A group-wise experiment was conducted with zebrafish (n = 83) swimming at U_opt_ for 6 h day^−1^ for 5 days week^−1^ for 4 weeks vs. zebrafish (n = 84) that rested during this period. Swimming zebrafish increased their total body length by 5.6% and body weight by 41.1% as compared to resting fish. For the first time, a highly significant exercise-induced growth is demonstrated in adult zebrafish. Expression analysis of a set of muscle growth marker genes revealed clear regulatory roles in relation to swimming-enhanced growth for genes such as growth hormone receptor b (*ghrb*), insulin-like growth factor 1 receptor a (*igf1ra*), troponin C (*stnnc*), slow myosin heavy chain 1 (*smyhc1*), troponin I2 (*tnni2*), myosin heavy polypeptide 2 (*myhz2*) and myostatin (*mstnb*).

**Conclusions/Significance:**

From the results of our study we can conclude that zebrafish can be used as an exercise model for enhanced growth, with implications in basic, biomedical and applied sciences, such as aquaculture.

## Introduction

Zebrafish has largely been accepted as a vertebrate multidisciplinary model for genetics, physiology, development, reproduction, disease (immunology, toxicology, oncology) and aging over recent years (recent reviews by [1]–[6]). Like more classical models such as rats and mice, zebrafish has a comprehensive genomic database. It also has many advantages for biological studies over rats and mice due to its shorter generation time, smaller size and because they are easier and cheaper to house. Especially for studies on reproduction and early development, the zebrafish has clear advantages over other model species because they are easy to reproduce, exhibit external fertilisation and have transparent embryos. However, in studies on exercise in relation to growth, the zebrafish has received relatively little attention.

Besides being a vertebrate multidisciplinary model, zebrafish is now also in the process of being accepted as a model for fish in aquaculture. A discussion is ongoing whether zebrafish exhibits indeterminate growth like in salmonids [7] or determinate growth like in mammals [8]. In salmonid species both hyperplasia (muscle growth by cell division) as well as hypertrophy (muscle growth by cell size) occur continuously during adulthood, in contrast to mammals that show only hypertrophy occurring during adulthood (reviewed by [9]). In teleosts that exhibit indeterminate growth such as salmonids, growth is stimulated by exercise [10]–[14] but a very limited amount of information is available on the mechanism(s) by which exercise potentiates growth.

Exercise-induced growth for salmonid species is optimal at specific speeds, most likely near optimal swimming speeds (U_opt_) where the cost of transport (COT, energy spent on swimming a certain unit of distance) is lowest and the energetic efficiency highest. At swimming speeds lower than U_opt_, a substantial amount of energy is lost due to higher spontaneous activity (e.g. aggression), while at speeds higher than U_opt_ swimming becomes unsustainable, stressful and the ensuing anaerobic metabolism will increase lactate levels, create an oxygen debt and finally cause fatigue (reviewed by [14]). Not only fuelling exercise but also growth rate, food conversion efficiency, lipid metabolism, protein turnover and aerobic capacity are therefore assumed to have a specific speed window and to be optimal at U_opt_.

Only a few previous studies have addressed the issue of swimming performance of adult zebrafish. Plaut and Gordon [15] reported on oxygen consumption rates during group-wise swimming. Endurance performance was high as shown by a low cost of swimming and the capacity of fish to swim at 0.40 m s^−1^ (13.0 body-lengths per second; BL s^−1^) for 2 h. However, the applied experimental system in this study did not allow higher swimming speeds, thus group-wise U_opt_ and critical swimming speed (U_crit_) could not be determined. In a later study, Plaut [16] measured U_crit_ of individual wild-type zebrafish at 0.560±0.048 m s^−1^ or 15.5 standard BL s^−1^. However, U_opt_ values for individually or group-wise swimming adult zebrafish are still unknown.

In this study we have therefore performed individual swimming experiments to quantify swimming economy by measurements of oxygen consumption to determine the U_opt_ in adult zebrafish. A large scale group-wise experiment at the determined U_opt_ was performed to demonstrate the effects of exercise on growth in adult zebrafish. Regulation of potential muscle growth effects was demonstrated by quantifying the expression of fourteen muscle growth marker genes.

## Materials and Methods

### Ethics

Experiments complied with the current laws of the Netherlands and were approved by the animal experimental committee (DEC number 09161).

### Experimental fish and conditions

Zebrafish, *Danio rerio* (n = 177), were purchased from a local pet shop (Selecta, Leiden, The Netherlands). Although their exact age was unknown, similarly sized zebrafish are found approximately at 60 days post fertilisation [17] and are not yet reproductively active. Fish were housed in fresh water at 28°C at a photoperiod regime of 16L:8D before and during the experiments. Fish were fed twice per day (DuplaRin pellets, Dupla, Gelsdrof, Germany) before and after experimental trials.

### Oxygen consumption measurements

Oxygen levels were measured using oxygen electrodes (Mettler Toledo, Tiel, The Netherlands) that were calibrated before use with sodium sulphite (0% Air Saturation, AS) and air (100% AS). During the swimming fitness experiment, oxygen was measured every 2 s. From the decline of the O_2_-concentration after closure of the water-inlet, the O_2_ consumption rate was calculated following the formula:



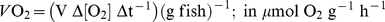
where Δ[O_2_]Δt^−1^ is the decrease of the O_2_ content in µmol h^−1^, and V is the water volume of the swimming tunnel (in L).

The percentages of decrease in oxygen content over time were calculated on the basis of solubility of oxygen under the given conditions in µmol O_2_, then expressed in µmol O_2_ h^−1^ and per g fish.

### Experimental set-up and protocol for demonstration of individual swimming fitness characteristics

**Figure 1:**
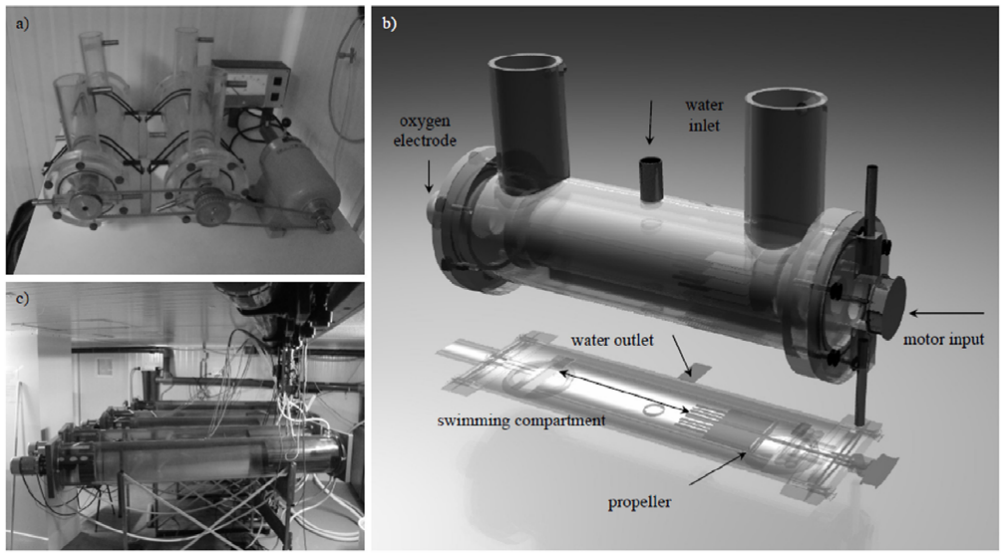
Small and large Blazka-type swimming tunnels. a) Set-up of the two small swimming tunnels used in the swimming fitness experiment with a volume of 1.8 L each, both driven through a belt by a motor (Multifix M80, Germany) connected with an ammeter (H.G.L., Japan). The relation between the water speed in the swimming chamber (V) in cm s^−1^ and the electric current (S) in micro-amperes (µA) was described by the formula V = 0.3257S-1.238. b) 3D design of the small swimming tunnel showing the inner and outer tubes, the locations of motor input, water inlet, water outlet, the oxygen electrode (Mettler Toledo, Tiel, The Netherlands) and the swimming chamber just behind a grid to create a laminar flow. c) 5 large 127 L swimming tunnels as described by van den Thillart et al. [22] of which two were used in a separate recirculation set-up housing the resters and swimmers during the long term training experiment.

Before introduction into the swim-tunnels, fish (n = 10) were anesthetized with clove oil (dissolved in 100% ethanol and used at a concentration of 0.5 mL in 0.5 L water) and measured for standard body length (BL, cm) and body weight (BW, g). For determination of U_crit_ at which fish fatigue (for review see [18]), individual fish (n = 10; 3.05±0.04 cm BL or ∼3.60 cm total body length (TL); 0.43±0.03 g BW) were placed in a set-up of the two small Blazka-style swimming respirometers of a volume of 1.8 L (Fig. 1a and b). Velocity was set at 0.05 m s^−1^. At this speed, the fish orientates itself against the current and swims steadily. Each respirometer was supplied with water at an approximate rate of 3 L min^−1^ (total volume of the recirculating system was approximately 150 L).

These conditions were maintained overnight to allow the fish to acclimate. Beginning about 20 h later, velocity was increased with increments of 0.05 m s^−1^ at 10 min intervals, until the fish fatigued. Fatigue was determined as the point when a fish could no longer maintain its position against the current and was swept against a mesh screen downstream of the tunnel and could not remove itself for three seconds (video S1). Visual observation ensured that no erratic swimming behaviour or premature fatigue occurred during the swimming trial. The speed was then briefly reduced to allow the fish to resume swimming. This process was repeated until fish fatigued for a third time and the test was terminated [19]. U_crit_ was calculated according to the equation:
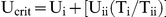
with U_i_ being the highest velocity maintained for the whole interval, U_ii_ the velocity increment (here: 0.05 m s^−1^), T_i_ the time elapsed from the start of the increment at fatigue velocity, and T_ii_ the interval time (here: 600 s; [20]). The absolute values (m s^−1^) were converted to relative swimming speeds in standard body lengths per second (BL s^−1^).

The fish were allowed to recover for a period of 2 hours from the U_crit_ experiment in water circulating at a velocity of 0.05 m s^−1^. Respiration measurements were performed on the following morning. Oxygen consumption was determined at a minimal flow of 5% U_crit_ for the measurement of RMR, routine metabolic rate, i.e. the oxygen uptake rate at routine swimming activity. Subsequently, oxygen consumption was measured at 25, 50, 75 and 100% U_crit_ in random order to avoid a habituation effect on the results. After 20 minutes at one speed, respirometers were reconnected to the continuous flow of water, which was saturated with air and the fish were given 30 min to recover. Subsequently, the measurement of *V*O_2_ was repeated at a different swimming speed. The resulting oxygen consumption rates were plotted against swimming speeds in % U_crit_. They were plotted polynomially by *V*O_2_  =  SMR + aU^2^ +bU, with SMR the standard metabolic rate in µmol O_2_ g^−1^ h^−1^, U the swimming speed in cm s^−1^ and a and b being constants, for the estimation by extrapolation of the SMR.

The cost of transport (COT; µmol g^−1^ m^−1^) was determined by dividing *V*O_2_ values by the corresponding U values. The U_opt_ (i.e. the speed at which the lowest oxygen uptake per unit distance swum, or lowest COT, occurred) was determined according to Palstra et al. [21]. The polynomial function of COT vs. speed was calculated and the lowest COT was found where the first derivative equals zero. The resulting values are the U_opt_ with a corresponding COT at U_opt_ (COT_opt_).

### Experimental set-up and protocol for group-wise long-term training

Two Blazka-type 127-L swim-tunnels as described by van den Thillart et al. [22] were used for demonstration of group-wise long-term training effects (Fig. 1c). The swim-tunnels were placed in a climatized room of about 100-m^2^. The total water content of about 500-L was recirculated continuously over a bio-filter.

Before introduction into the swim-tunnels, fish (n = 167) were anaesthetized as described before and measured. Randomly selected fish were introduced in one swim-tunnel for swimming (n = 83; 3.59±0.02 cm TL, 0.41±0.01 g BW) and in another swim-tunnel for resting (n = 84; 3.60±0.02 cm TL, 0.42±0.01 g BW). When introducing the fish in the tunnel for resting, five fish were damaged by entering the stream grid and were removed from the experiment leaving 79 fish in the tunnel for resting.

Temperature and light conditions in the swim-tunnels were identical as before introduction in the swim-tunnels. Fish were again fed twice per day by injecting food portions in the circulation pump. Fish sensed the food entering the system and immediately gathered at the front of the tunnel near the grid where they fed as soon as the food entered the tunnel. Fish were kept in a resting condition at a flow rate of 0.1 m s^−1^ to ensure sufficient mixing of the water (including food and O_2_) in the tunnel. At this flow, fish moved freely through the tunnel in all directions. Fish were acclimatised for four days until the start of the experiment at day 5 (Monday).

The long-term training protocol involved sustained swimming at the U_opt_ for 6 h per day (10.00–16.00 h), for 5 days per week lasting for 4 weeks. The motor speed was increased slowly in one tunnel up to U_opt_ while the motor of the other tunnel was maintained at the flow rate of the resting condition. All fish were fed before and after these 6 h training periods.

After 20 experimental days, fish were removed from the tunnels and were anaesthetized as described before, counted and measured again. Muscle tissue was dissected dorsally from the lateral line in the epaxial quadrant and preserved in RNA*later* (Ambion) at −20°C.

### Primer sequences and relative quantitative PCR (Q-PCR)

Primer sequences and GenBank accession numbers of the targeted genes are given in Table 1. Primers for growth hormone receptors a and b (*ghra*, *ghrb*), insulin-like growth factor 1 (*igf1*), insulin-like growth factor 1 receptors a and b (*igf1ra*, *igf1rb*), zebrafish target of rapamycin (*ztor*), forkhead box O5 (*foxo5*), slow-specific troponin C (*stnnc*), slow myosin heavy chain 1 (*smyhc1*), fast muscle troponin I (*tnni2*), myosin heavy polypeptide 2 fast muscle specific (*myhz2*), myostatin b (*mstnb*) and myogenin (*myog*) were designed using the Genamics Expression software (www.genamics.com). Primers for 40S ribosomal protein S18 (*rps18*) were published by McCurley & Callard [23] (Table 1a) or also designed (Table 1b). Primers for peroxisome proliferator-activated receptor γ coactivator 1α (*pgc1α*) were published by by LeMoine et al [24].

**Table 1:** Nucleotide sequence and GenBank accession number of primers used for Q-PCR.

	Target gene	Genbank	Forward sequence (5′-3′)	Reverse sequence (5′-3′)
A)	*rps18*	BX296557	TCGCTAGTTGGCATCGTTTATG	CGGAGGTTCGAAGACGATCA
	*ghra*	BC134903	TCCTCCTTCATCGCTGCCTAT	GCAAAGGCTGATAGAAAGGAAACA
	*ghrb*	EU649775	CAGAAGGCAGCAGCGAAGAC	CGGTGTTGGCTAAGCAGTCAGA
	*igf1ra*	AF400275	GTTTGACGAGACGCAGCCTTAC	CAAAGGGAGGAGGGAAATGTGT
	*igf1rb*	AF400276	CGGATGCGTCGGATGTGTGT	GTCTGGCGGAAATAACAAATGAGT
	*igf1*	BC114262	CGAGCACAACGACACACAGATATT	CCAGTCTTTCTTTCTTCCCCTCTT
	*ztor*	DQ666026	GCCATTCAGATCATCAACCGAGT	GCGATGCCGATTCCCACTCT
	*foxo5*	AF114262	GCTGTTAGTCTGAATCCTGTGGGA	GCAGACAGCAAACTTGGCAAA
	*pgc1α*	AY998087	TGAGGAAAATGAGGCCAACT	AGCTTCTTCAGCAGGGAAGG
B)	*rps18*	AY099517	TGAGGTTGAGAGGGTGGTGACTA	CCTTCTGTCTGTTCAGGAACCAGT
	*stnnc*	BC071546	GCAAGATCGACTACGACGAGTTCT	AGGCAGCATTGGTTCAGGGA
	*smyhc1*	AY921649	GAGCCGTGATTCAGGACCCA	TGGCTTCACAACAAACAAACTCGT
	*tnni2*	AF425744	GGTATGGACGGCAGGAAGAAGA	GGAACGGGAGGTTTACAGGACAG
	*myhz2*	BC071279	GCCTGAGCTGATTGAAATGACGC	GCTCCTCACGCTGCTTCTGCTT
	*mstnb*	AF540956	AGACCGCTGTGGCTGCTCAT	GGTAATGTATGGTGGGTGGTGGA
	*myog*	AF202639	AGTGGACAGCATAACGGGAACAG	GCTGGTCTGAAGGTAACGGTGAG

Primers used for housekeeping gene *rps18* were different for target genes measured under (A) and (B).

RNA was isolated with TRIzol (Invitrogen, Baro, Spain), DNAse treated with RQ1 DNAse (Promega, Madison, USA) and reverse transcribed using Superscript III (Invitrogen, Baro, Spain), according to the manufacturers' protocols. For the quantification of mRNA expression, real-time quantitative PCR (Q-PCR) was performed. cDNA was diluted 1∶10 or 1∶25 for target genes and 1∶25 or 1∶2000 for *rps18*, and used as a template. The reactions (20 µl final volume) contained 10 µl of SYBR GreenER qPCR SuperMix (Invitrogen), 500 nM concentration of forward and reverse primers and 5 µl of cDNA. Reactions were run in a iCycler Thermal Cycler (BioRad) using the following protocol: 2 min at 50°C, 8 min at 95°C, followed by 40 cycles of 15 sec denaturation at 95°C and 30 sec at the corresponding melting temperatures, and a final melting curve of 81 cycles from 55°C to 95°C (0.5°C increments every 10 sec). Samples were run in triplicate and fluorescence was measured at the end of every extension step. Fluorescence readings were used to estimate the values for the threshold cycles (Ct).

The Ct values were normalized for each gene against those obtained for the housekeeping gene *rps18*. The expression of *rps18* was relatively low but stable and the very limited individual variation was extremely consistent. There were no differences in *rps18* expression levels between the two groups. The measured expression of target genes was therefore not impacted by differences in *rps18* expression.

### Analysis and statistics

All statistical analyses were performed with SPSS 16.0. Differences with P≤0.05 were considered significant. Values are expressed as average ± standard error in all cases. Normal distribution in any case was first checked by Kolmogorov Smirnoff tests. Size differences in TL and in BW between the two groups (‘swimmers’ vs ‘resters’) at the start of the experiment were checked with unpaired two-tailed student t-tests and found to be absent. Size changes in TL and in BW over the course of the experiment (pre-experimental vs. post-experimental values) were checked within swim- and rest-groups with paired one-tailed student t-tests. Finally, size differences in TL and in BW between the two groups (‘swimmers’ vs ‘resters’) at the end of the experiment were checked with unpaired one-tailed student t-tests.

Size differences in TL and in BW between the swimmers (n = 8) and resters (n = 8) that were examined by Q-PCR were again checked with unpaired one-tailed student t-tests and were still significantly different (P<0.05). Four outliers [25] in the expression of *pgc1α* (1), *myhz2* (1) and *mstnb* (2) were removed from data. Normalized *C*t values for each of the growth marker genes were expressed as fold changes (fc) using the relative quantification method [26], calculated for swimmers relative to resters and compared between both groups performing Mann-Whitney U-tests.

## Results

### Swimming economy

**Figure 2:**
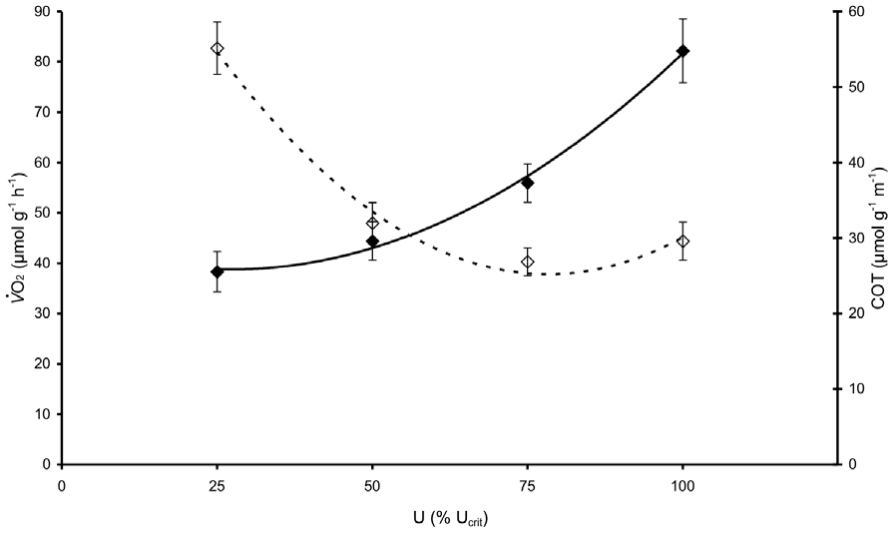
Swimming economy of zebrafish. Oxygen uptake (*V*O_2_; black diamonds, solid line, average ± standard error, n = 10) at 25, 50, 75 and 100% U_crit_ (U) with a U_crit_ of 0.548±0.007 m s^−1^ or 18.0±0.2 BL s^−1^. The curve followed the polynomial equation: *V*O_2_  =  SMR +0.0081U^2^ - 0.4353 U (r^2^ = 0.99) with an SMR extrapolated to zero swimming speed of 43.79±3.39 µmol g^−1^ h^−1^. Cost of transport (COT; white diamonds, broken line, average ± standard error, n = 10) at 25, 50, 75 and 100% U_crit_ (U). The curve followed the polynomial equation: COT  = 0.0104 U^2^ – 1.6239 U +88.767 with the lowest value of 25.23±4.03 µmol g^−1^ m^−1^ at U_opt_ of 0.396±0.019 m s^−1^ (72.26±0.29% U_crit_).

Individual zebrafish were able to swim at a U_crit_ of 0.548±0.007 m s^−1^ or 18.0±0.2 BL s^−1^ (Figure 2; video S1). SMR was calculated at 43.79±3.39 µmol g^−1^ h^−1^ and RMR was slightly lower at 38.13±5.31 µmol g^−1^ h^−1^ with high individual variation. Oxygen consumption at 25% U_crit_ was still similar to RMR but then increased up to a maximal oxygen consumption at U_crit_ of 82.18±6.50 µmol g^−1^ h^−1^with increasing speed. Plotting the COT values polynomially was possible at high r^2^ (>0.99) allowing to precisely determine an optimal swimming speed (U_opt_) of 0.396±0.019 m s^−1^ or 13.0±0.6 standard BL s^−1^, at 72.26±0.29% of U_crit_. The cost of transport at optimal swimming speed (COT_opt_) was 25.23±4.03 µmol g^−1^ m^−1^. The determined U_opt_ was applied as swimming speed during the long-term training experiment.

### Swimming behaviour during long-term training

Swimmers displayed a typical burst-and-glide swimming pattern as observed in other cyprinids like carp [19]. At the U_opt_, however, the speed was already too fast for gliding and bursts were almost continuous as shown by the tail beat action (video S2). Fish were checked regularly but they did not fatigue, staying in the flow all the time. Mortality during the whole experimental time was very low among swimmers (n = 5 or 6.0%) and not different from resters (n = 5 or 6.4%) indicating that no fish were lost because of the exercise protocol.

### Swimming-induced growth

**Figure 3:**
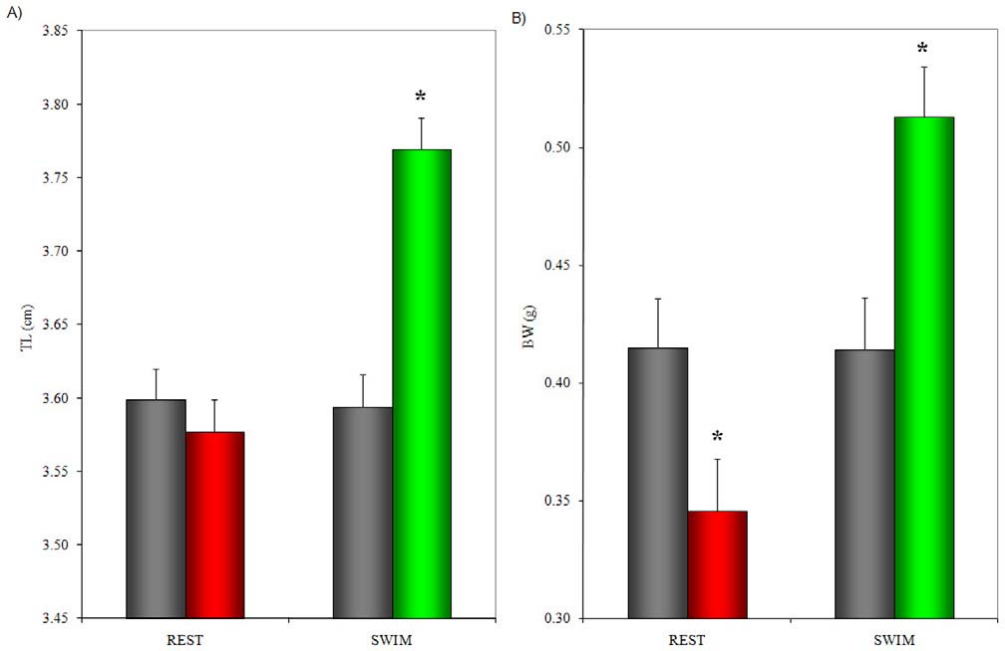
Growth (average ± standard error) over 4 weeks of swimming and resting fish. a) Difference in total body length (cm). b) Difference in body weight (g). Length and weight of swimmers increased significantly in comparison to the resters as well as to the controls. Resters decreased their BW compared to the controls (grey bars: before experiment, coloured bars: same fish after experiment). The significant differences indicated by asterisks involve both differences between post-experimental values and pre-experimental values as well as between post-experimental values of swimmers and resters.

Total length and weight of the swimmers increased in a highly significant manner (from 3.59±0.02 to 3.77±0.02 cm TL, P<10^−7^: from 0.41±0.01 to 0.51±0.01 g BW; P<10^−7^) during the experimental period (Fig. 3). In contrast, the resters did not show an increase in total length and weight but instead a decrease in body weight (from 0.42±0.01 to 0.35±0.01 g BW; P<10^−5^). Differences in both TL (P<10^−8^) and BW (P<10^−16^) between swimmers and resters at the end of the experiment were highly significant, with swimmers increasing TL and BW by 5.6% and 41.1%, respectively, over resting fish.

### Muscle growth marker gene expression

**Figure 4:**
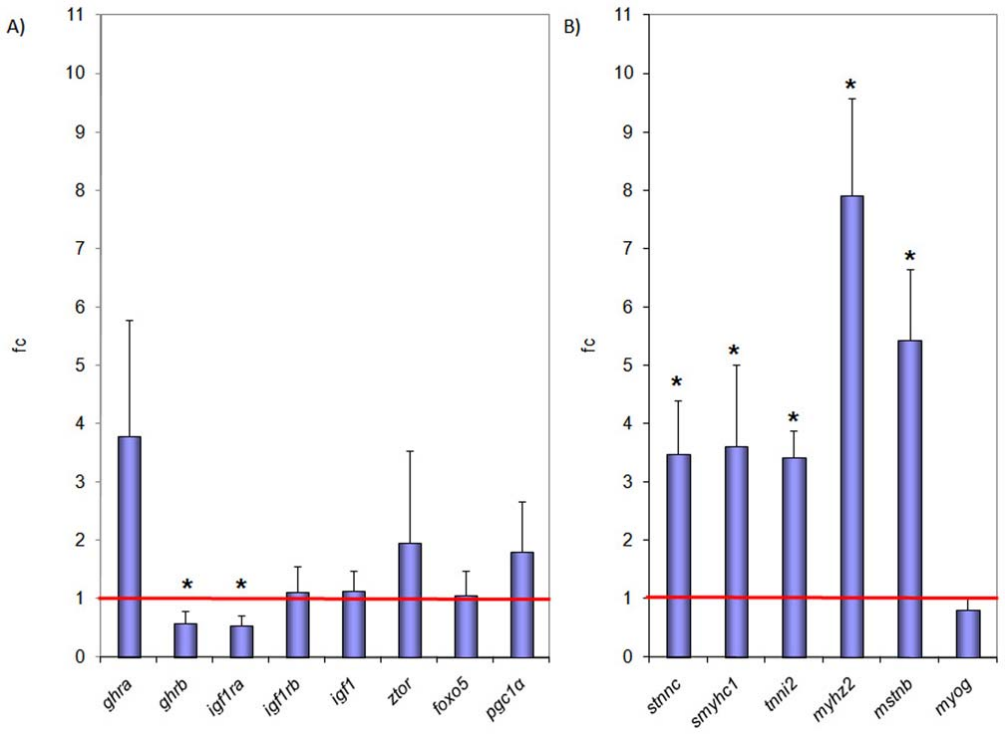
Group-wise comparison of marker gene expression. a) Expression of growth marker genes *ghra*, *ghrb*, *igf1ra*, *igf1rb*, *igf1*, *ztor*, *foxo5* and *pgc1α* and b) *stnnc*, *smyhc1*, *tnni2*, *myhz2*, *mstnb* and *myog* (*see text for abbreviations*) normalized for the expression of *rps18* represented as fold change in swimmers (n = 8) over resters (n = 8). Significant differences (P<0.05) are indicated by *. Expression of *ghrb* and *igf1ra* was down-regulated and expression of *stnnc*, *smyhc1*, *tnni2*, *myhz2* and *mstnb* was up-regulated in swimmers in comparison with the resters.

In order to decipher the molecular changes taking place in the skeletal muscle of fish experiencing increased growth as a result of swimming exercise, we examined the expression levels of a set of known marker genes for muscle growth in swimmers and resters. These marker genes included *ghra*, *ghrb*, members of the IGF-1/PI3K/AKT pathway such as *igf1*, *igf1ra*, *igf1rb* and *ztor*, as well as *foxo5* and *pgc1α*. Additionally, expression of muscle growth markers involved in myofibre developmental regulation (*myog*), differentiation (*mstnb*) and sarcomeric structure (*stnnc*, *smyhc1*, *tnni2*, *myhz2*) was examined. Group-wise comparison of swimmers relative to resters showed a significant down-regulation of the expression of *ghrb* (fc 0.58±0.20) and *igf1ra* (fc 0.53±0.18) (Fig. 4a). The expression levels of *ghra*, *igf1rb*, *igf1*, *ztor*, *foxo5* and *pgc1α* were not significantly different between swimmers and resters. Expression of *stnnc* (fc 3.47±0.93), *smyhc1* (fc 3.60±1.42), *tnni2* (fc 3.42±0.45), *myhz2* (fc 7.92±1.65) and *mstnb* (fc 5.44±1.20) were significantly up-regulated (Fig. 4b) while *myog* expression was not different between swimmers and resters.

## Discussion

The results of our study contribute to establishing zebrafish as a model for exercise-induced growth. In this study we report on the characteristics of individual swimming economy of adult zebrafish and set the basis for the establishment of exercise protocols for this species. By applying a 20-day training protocol at optimal swimming speed, we have shown for the first time a highly significant exercise-induced growth in adult zebrafish. Furthermore, muscle expression of several growth marker genes was clearly regulated in relation to swimming-enhanced growth.

We have demonstrated that zebrafish swim at an optimal speed U_opt_ of 0.396±0.019 m s^−1^ or 13.0 standard BL s^−1^. Such swimming speed seems remarkably high in comparison with values for other fish species. In twenty-two fish species that have been examined, U_opt_ values were as high as 5.8 BL s^−1^ (reviewed by [27]) and the U_opt_ for a similarly sized cyprinid (*Rutilus rutilus)* was 3.7 BL s^−1^ [28]. The endurance capacity of zebrafish is reflected by the oxygen consumption levels that are among the highest measured in similarly sized fish at similar temperatures [29]. In our study, U_opt_ was found at 72.3% of the U_crit_. U_crit_ was determined at 0.548±0.007 m s^−1^ or 18.0 standard BL s^−1^ (Fig. 2, video S1), similar as reported by Plaut [16]. The burst and glide swimming mode observed at U_opt_ indicates that this speed may be powered by both aerobic and anaerobic muscle action. The low *V*O_2_ values at U_opt_ reflect only the aerobic component of the swimming economy. The U_opt_, which is the energetically most efficient swimming speed, was then applied during long term training experiments of adult zebrafish. During these training trials of 6 h day^−1^ swimming at U_opt_ for 20 days, fish did not show any sign of fatigue (also video S2).

Swimming for only 20 days at U_opt_ increased total body length by 5.6% and body weight by 41.1% vs. resting fish. This increase in body growth in zebrafish under exercise conditions is even higher than that reported for adult salmonid fish. For example, brook trout (*Salvelinus fontinalis)* yearlings swimming at U_opt_ for 20 days increased 3.5% in BL and 34% in BW [10] and adult Atlantic salmon (*Salmo salar*) exposed to 8 months of sustained swimming showed a 38% increase in growth with respect to the non-exercised fish [13]. Zebrafish thus shows comparable swimming-enhanced growth to commercially important species like trout and salmon. Zebrafish could therefore be used as a growth model for aquaculture, since exercise represents a natural way of stimulating growth with major potential economic benefits.

Our results on the growth stimulatory effects of exercise in zebrafish are in contrast with earlier studies that indicated that exercise-enhanced growth occurs only early in development (21–24 days post fertilisation; [17]) and is absent in adult fish [30]. This discrepancy might be related to the lower exercise intensity applied in these other studies. McClelland et al. [30] examined muscle gene expression in adult zebrafish swimming with a similar protocol to ours but at lower increasing speeds from 2 to 5 BL s^−1^ with increments of 1 BL per week. LeMoine et al [24] applied the same protocol and then continued up to 8 weeks at a swimming speed of 10 BL s^−1^. Suboptimal speeds like these might however not reflect optimal muscle growth conditions, which are assumed to be optimal at U_opt_. While others applied relative low exercise levels, in this study we exercised the experimental fish at their optimal swimming speed.

Earlier studies considered zebrafish growth as determinate like in mammals [8], [31]. Reports in the literature indicate that growth of white muscle by hyperplasia stops in zebrafish at ∼20 mm TL while hypertrophy continues into adulthood [17], [32]. In salmonids, growth induced by training is likely a combination of both [14], [33]. The comparable growth rate in adult zebrafish observed in this study makes this species an interesting exercise model. Muscle morphological, biochemical and transcriptomic analyses of zebrafish exposed to long-term swimming should provide more conclusive answers about whether this species experiences determinate growth like mammals or indeterminate growth like most larger teleost fish.

The molecular regulation of swimming-enhanced muscle growth was studied by quantifying the expression of fourteen muscle growth marker genes after long-term training. Swimming caused a significant increase in the expression of four genes that encode proteins involved in sarcomeric structure: two components of the troponin complex (*stnnc* and *tnni2*) and two myosins (*smyhc1* and *myhz2*). Increased expression of these genes in exercised zebrafish indicates that swimming exercise may induce molecular mechanisms that participate in the regulation of striated muscle contraction. Interestingly, up-regulation of the expression of fast fibre markers (*tnni2* and *myhz2*) was accompanied by parallel changes in the expression of slow fibre markers (*stnnc* and *smyhc1*). As previously suggested by Van der Meulen et al [17], increased expression of slow fibre markers in epaxial skeletal muscle of exercised zebrafish could evidence a shift towards a slow aerobic phenotype. LeMoine et al [24] also recently reported an increase in the aerobic capacity of white muscle in adult zebrafish in response to long-term swimming exercise that was not correlated with increased expression of *pgc1α*, known in mammals to induce production of mitochondria and a shift to slow-twitch oxidative fibres (reviewed by [34]). In line with these reports, no significant effects of exercise training in zebrafish were observed on *pgc1α* expression in the present study. Furthermore, we observed significant changes in the expression of *ghrb*, *igf1ra* and *mstnb*, known regulators of muscle growth in fish including zebrafish [35]–[37]), at the termination of the growth-stimulating training regime. In swimmers, the decreased expression of *ghrb* and *igf1ra*, genes encoding receptors for growth hormone and IGF-I, two well-known growth-stimulatory factors [38], [39], and the increased expression of *mstnb*, a negative regulator of growth [37], suggest the activation of mechanisms controlling growth under growth-stimulatory conditions. In support of this hypothesis, muscle compensatory growth induced by refeeding also results in a decrease in the expression of *ghr* and *igfra*, as shown in several teleost species [40]–[43]. However, although other studies have not observed increases in the expression of *mstnb* in the muscle of exercised zebrafish [17] or rainbow trout [44], the significant up-regulation in the expression of *mstnb* in swimmers is in accordance with the lack of change in myogenin expression under the conditions employed in the present study. In fact, myostatin may inhibit myogenin expression in zebrafish, as evidenced by the increased expression of myogenin in embryos in which myostatin was knocked-down with antisense morpholinos [45]. Therefore, swimming-enhanced muscle growth in adult zebrafish is characterized by the increased expression of genes involved in muscle contraction and, importantly, by changes in the expression of key growth marker genes suggesting suppression of growth at least at the end of the growth period. It is important to consider that the measurement of gene expression in this study represents the situation at a single moment in time, i.e. at the end of the training experiment when significant growth enhancement had occurred. We hypothesize that the expression of the genes involved in muscle growth may have a more dynamic nature than expected and that a time series of measurements may provide additional insights regarding temporal changes in gene expression. In general, it can be argued that up-regulation of the expression of genes that are involved in the initiation of a particular process may occur at the start of that process (i.e. enhanced growth in this study) and that, at a later time, expression is decreased due to negative feedback mechanisms. The recent study by LeMoine et al [24] in the muscle of exercising zebrafish would seem to support this hypothesis. Furthermore, a similar down-regulation of a large number of genes associated with growth was observed in skeletal muscle of exercised rainbow trout by RNA-seq (Palstra and Planas, unpublished data). Additional supporting evidence comes from mammalian studies under opposite conditions of long-term muscle denervation [46] and fasting [47] where genes involved in protein synthesis are up-regulated.

Although changes in the expression of growth marker genes evidence important changes in pathways known to be involved in growth regulation, it is clear that swimming-enhanced growth in skeletal muscle is a multifactorial process regulated most likely at various levels (mRNA, protein, enzyme activities, etc). High-throughput analyses may therefore be instrumental in elucidating the molecular and cellular changes taking place in muscle in response to exercise.

### Perspectives of the zebrafish exercise model

In conclusion, swimming economy of individual adult zebrafish was characterised for the first time. Our study indicates that zebrafish can be used as an exercise model for enhanced growth, with implications in basic, biomedical and applied sciences, such as aquaculture.

Not only for studying muscle growth would zebrafish be an appropriate exercise model. Zebrafish has been recently recognized as a valuable model for immunological studies [48] since adult zebrafish posses an adaptive and innate immune system similar to mammals [49]–[53]. Given the beneficial effects of exercise on mammalian immune function [54]–[56], zebrafish can be used as an exercise model to investigate exercise-induced stimulation of immune function and fast large scale screening for the potential beneficial effects of exercise on various diseases. Current studies in our laboratory are devoted to test the hypothesis that fish subjected to the experimental protocol described in this study may have a differential transcriptomic response to an immune challenge. This would represent a first step towards using a tractable experimental model to deepen our understanding of exercise-induced enhancement of immune function in vertebrates, including humans.

## Supporting Information

Video S1 This movie shows two zebrafish swimming near U_crit_ synchronously in the small swim-tunnels that were used for the individual swimming fitness experiment (note that blue light was not used during the actual experiment and oxygen electrodes were connected at the back side). After switching to a higher speed, the fish in the first tunnel fatigues and is dragged against the back screen by the stream. Zebrafish have a critical swimming speed U_crit_ of 0.548 ± 0.007 m s^−1^ or 18.0 ± 0.2 standard BL s^−1^.
(4.69 MB WMV)

Video S2 This movie shows the behaviour of zebrafish swimming at U_opt_ (swim-tunnel foreground) and resting fish (swim-tunnel background) during the group-wise long-term training experiment in Blazka-type 127 L swim-tunnels. Swimmers displayed a typical burst-and-glide swimming pattern. The optimal swimming speed U_opt_ however is so fast that there is not much time for gliding and bursts are almost continuous as shown by the constant tail beats. Zebrafish have an optimal swimming speed at 72.3% U_crit_ of 0.396 ± 0.019 m s^−1^ or 13.0 ± 0.6 standard BL s^−1^.
(1.24 MB WMV)
